# Prevalence and patterns of illicit drug use in people living with HIV in Spain: A cross-sectional study

**DOI:** 10.1371/journal.pone.0211252

**Published:** 2019-06-17

**Authors:** Maria Jose Fuster-RuizdeApodaca, Vanessa Castro-Granell, Noé Garin, Ana Laguía, Ángeles Jaén, Carlos Iniesta, Santiago Cenoz, María José Galindo

**Affiliations:** 1 Spanish Interdisciplinary Aids Society (Sociedad Española Interdisciplinaria del Sida, SEISIDA), Madrid, Spain; 2 Doctoral Programme in Pharmacy, Granada University, Granada, Spain; 3 Department of Pharmacy, Hospital Marina Baixa, Villajoyosa, Alicante, Spain; 4 Department of Pharmacy, Hospital Santa Creu i Sant Pau, Universitat Autònoma de Barcelona, Barcelona, Spain; 5 Instituto de Salud Carlos III, Centro de Investigación Biomédica en Red de salud Mental (CIBERSAM), Madrid, Spain; 6 School of Health Science Blanquerna, Universitat Ramon Llull, Barcelona, Spain; 7 Department of Social and Organizational Psychology, Facultad de Psicología, Universidad Nacional de Educación a Distancia (UNED), Madrid, Spain; 8 Research Unit, Research Foundation MútuaTerrassa, Universitat de Barcelona, Barcelona, Spain; 9 Centro Nacional de Epidemiología, Instituto de Salud Carlos III, Madrid, Spain; 10 Medical Department, ViiV Healthcare, Tres Cantos, Madrid, Spain; 11 Department of Infectious Diseases, Hospital Clínico Universitario, Valencia, Spain; Rutgers School of Public Health, UNITED STATES

## Abstract

This study assessed the prevalence and patterns of drug use among people living with HIV (PLHIV) in Spain. We conducted an observational cross-sectional study including 1401 PLHIV. Data were collected through 33 sites across Spain using an online computer-assisted self-administered interview. The survey measured use of illicit drugs and other substances, treatment adherence and health-related variables. To analyse patterns of drug use we performed cluster analysis in two stages. The most frequently consumed substances were: alcohol (86.7%), tobacco (55.0%), illicit drugs (49.5%), other substances (27.1%). The most prevalent illicit drugs used were cannabis (73.8%), cocaine powder (53.9%), and poppers (45.4%). Results found four clusters of PLHIV who used drugs. Two of them were composed mainly of heterosexuals (HTX): Cluster 1 (n = 172) presented the lowest polydrug use and they were mainly users of cannabis, and Cluster 2 (n = 84) grouped mostly men who used mainly heroin and cocaine; which had the highest percentage of people who inject drugs and presented the lowest level of treatment adherence (79.8±14.2; *p* < .0001). The other two clusters were composed mainly of men who have sex with men (MSM), who were mostly users of recreational drugs. Cluster 3 (n = 285) reported moderate consumption, both regarding frequency and diversity of drugs used, while Cluster 4 (n = 153) was characterized by the highest drug polyconsumption (7.4±2.2; *p* < .0001), and 4 grouped MSM who injected recreational drugs, and who reported the highest frequency of use of drugs in a sexual context (2.6±0.8; *p* < .0001) and rates of sexually transmitted infections (1.8±1.1; *p* < .01). This is the largest multi-centre cross-sectional study assessing the current prevalence and patterns of drug use among PLHIV in Spain. The highest prevalence of drug use was found among MSM, although HTX who used heroin and cocaine (Cluster 2) had the most problems with adherence to HIV treatment and the worst health status.

## Introduction

The human immunodeficiency virus (HIV) continues to be a major public health challenge in Spain, with 145,000 people living with HIV (PLHIV) in 2016 [1]. Among the new HIV cases that year, sexual transmission accounted for 79.6% of them, 53.1% of whom corresponded to men who have sex with men (MSM) and 26.5% to heterosexuals (HTX), while only 3.6% were attributed to people who inject drugs (PWID) [1]. Regarding prevalence, HTX and PWID were still the largest subgroups of PLHIV, 32.5%, and 30.9% respectively, although their relative weight continued to decrease gradually in favour of MSM [2]. Both the effectiveness of the highly active antiretroviral therapy (ART) and the high rate of access to treatment − nine out of ten PLHIV in Spain − have led to a substantial mortality reduction over the last decades [3].

Despite the increased life expectancy for PLHIV, certain personal aspects may have an important impact on their health care and health-related quality of life. One of these aspects is substance use. A review on recreational drug use in PLHIV in Europe showed consumption prevalence above 50% in most studies, especially in those focusing on MSM. Prevalence ranged from 5.5 to 82.4% [4]. The ASTRA study found that 51% of 2,248 MSM PLHIV had used recreational drugs in the previous three months in the UK [5]. In Spain, the prevalence of illicit drug use in PLHIV remains unclear. The EMIS Survey, addressed to MSM only, found higher consumption of illicit drugs in those PLHIV using many different recall periods [6]. A recent study focusing on MSM with HIV in Madrid, Spain, found that 59% of them had used illicit drugs during the previous year [7]. A similar prevalence over the same period has been reported in the area of Catalonia, Spain [8]. With regard to individuals under treatment, a study focusing on PLHIV attending one hospital found a prevalence of use of 44.2% in the last 12 months [9].

Studies to date have reported higher drug consumption rates in PLHIV compared with the general population [10]. There is, however, considerable variability regarding the frequency of use of the different drugs in the literature [4]. The EMIS study showed that poppers (56.2%), cannabis (41.9%), cocaine (38.2%), sildenafil (32.9%), ecstasy (24.2%) and GHB/GBL (18.9%) were the substances most frequently consumed over the past 12 months by MSM living with HIV in Spain [11]. Some studies found that polydrug use, the use of various drugs over a predefined period, was about 50% in some cases [9].

Studies have failed to identify clear patterns of drug consumption because the list of drugs to be assessed varies significantly with time and across countries or social groups [9]. However, the literature has highlighted two important patterns of drug use [12]. The first one, ‘chemsex’, covers sexualized drug use and is practised mainly by MSM [12,13]. In the UK, the most common ‘chemsex’ drugs are crystal methamphetamine, GHB/GBL, mephedrone, and to a lesser extent, ketamine [14,15]. In Spain, according to the U-SEX GESIDA-9416 study, cocaine was the most frequent drug used in a sexual context, while crystal methamphetamine was the least frequent [7]. Also, studies suggested that the practice of injecting recreational drugs at sex parties − ‘slamming or slamsex’ − might be increasing in MSM [16]. The second pattern would involve ‘party drugs’, such as cocaine, GHB, ketamine and amphetamines [12].

In addition to these patterns, there are still PLHIV using traditional intravenous drugs, such as heroin or crack cocaine [6]. The literature on trends of drug use suggested that either fewer people are taking these drugs nowadays or users are switching to new drugs [17,18]. In Spain, both the prevalence of use of heroin and its intravenous use decreased markedly from the nineties to 2006 thanks to opioid substitution therapy, syringe exchange programmes and replacement by other non-intravenous administration routes [19]. To our knowledge, although the percentage of PLHIV using these drugs nowadays in Spain may be low, there are no studies on the current prevalence and patterns of use of these substances.

Substance use may have a negative impact on HIV care and prevention. Some literature showed a negative effect of illicit drug use on HIV disease [20]; on transmission risk behaviour leading to the acquisition of sexually transmitted infections (STIs) [7,11,16], low adherence to ART [21,22], and interactions with ART [23,24]. The first step to tackle this situation is to understand the current use of these drugs. Thus, this study aimed to assess the prevalence of drug use and to explore the patterns of consumption among PLHIV in Spain.

## Materials and methods

### Study design

This observational, cross-sectional study consisted of an electronic interview focusing on illicit drug use in PLHIV. The study was performed in Spain, including data from 12 Spanish Autonomous Communities between November 2016 and May 2017. The interview was offered to adults over 18 years old living with HIV under ART for at least one year. Participants with severe psychiatric or cognitive disorders were considered not eligible.

We used a community-based participatory research paradigm, involving members of the population under study in all research phases [25,26]. Also, a group of experts from several social and health-care fields guided the research and participated in its different stages.

The Ethics Committee of the Hospital Clínico de Valencia approved the research protocol. All procedures of the study followed the Helsinki Declaration principles, as well as the guidelines for good clinical practice.

### Procedures

The study was coordinated by the Spanish AIDS society (SEISIDA), which contacted professionals from 36 institutions across the country. A total of 33 sites agreed to collaborate in the study, 12 hospitals and 21 Non-Governmental Organizations (NGOs), which used convenience sampling for participants’ inclusion. Of the total sample, 51.7% of the individuals were recruited in NGOs.

The collaborating professionals included participants during their clinical visits or while attending to various services. Additionally, a few NGOs recruited participants by e-mail. The goals of the study were explained to potential participants, requesting their participation and obtaining their signed informed consent. An online computer-assisted self-administered interview (CASI) designed with Qualtrics was administered to participants with a tablet after providing appropriate instructions. Professionals were available to clarify any doubts or difficulties during questionnaire completion. The average time needed to complete the questionnaire was 40 minutes.

We calculated an initial sample of 1,500 PLHIV distributed among the different participating sites across Spain. We distributed the sample in order to: (1) maintain the distribution of the burden of HIV in the main Spanish regions, and (2) have an extensive geographical representation. S1 Table shows the number and percentage of surveys collected by the different Spanish regions and its HIV incidence in the last five years [1]. A total of 1,401 PLHIV agreed to participate and finished the online survey (data are available from 10.6084/m9.figshare.8059784). The final response rate was 93.4%, and it varied across sites ranging from 82 to 100%. There were lower rates in hospitals compared with NGOs. Main reasons for refusing to participate were lack of time, survey length, visual impairment or lack of skills to use tablets. Participants were compensated with 15 euros.

### Measures

We designed the survey according to the results of an earlier qualitative study and previous evidence in the literature, following the methodological recommendations on the construction of scales and wording of items [27,28]. The research team and the group of expert consultants collaborated in the design process. The questionnaire was previously piloted and refined using a sample of 61 PLHIV who used drugs. The survey contained the following variables:

#### Use of illicit drugs and other substances

**Fig 1:**
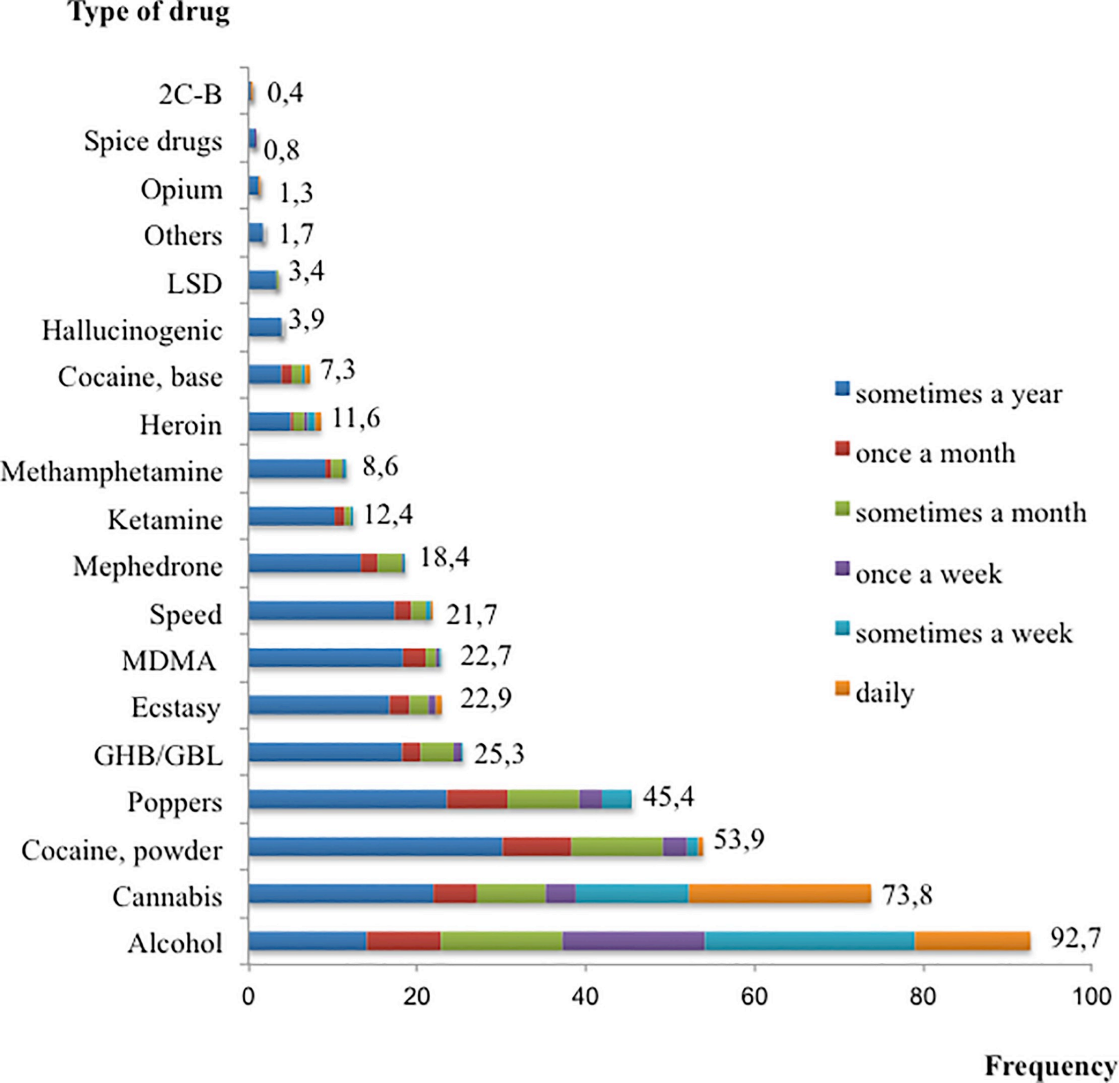
Prevalence and frequency of use of illicit drugs among PLHIV using drugs. N = 694.

The survey included a list of 18 illicit drugs (Fig 1), their frequency of use and their route of administration. Besides, it included questions asking participants about the frequency of alcohol use and smoking status. The survey also incorporated items related to the consumption of other substances or medicines: erection enhancers; methadone, morphine, and other opioids; anabolic steroids; sedatives or hypnotics, antidepressants. The frequency of use for all substances except tobacco was rated on a 6-point scale ranging from ‘sometimes in the last year’ to ‘daily’. Tobacco use was measured by the amount of cigarettes smoked per day.

We also included items related to the drug consumption context: (a) use of drugs previous to HIV diagnosis (yes/no); (b) main reasons for using drugs (8 reasons were listed and one open question); and (c) frequency of use of drugs in a sexual context. Two items were rated on a 4-point scale; these items were merged in the analyses due to the good correlation observed (r = .59).

#### Treatment adherence and other health-related variables

We used the Questionnaire to Evaluate the Adherence to HIV Therapy (CEAT-VIH) [29]. The 2.0 version of the scale is comprised of 17 items rated on a 5-point scale. Negative items were reverse-coded. We calculated a composite score, with higher scores indicating higher treatment adherence. The scale showed an adequate reliability in our study (Cronbach’s α = .78).

Moreover, the survey included the following questions related to participants’ health status: years living with HIV; CD4 cell count; viral load; and STIs in the last year.

Finally, the questionnaire collected several socio-demographic characteristics: age; gender; sexual orientation; level of education; employment status; financial resources; and city of residence.

### Data analysis

Frequencies, proportions, ranges, means, SDs and cross-tabulations were applied for descriptive analysis. To analyse the diverse typologies of PLHIV, we performed a two-step cluster analysis using demographics, health-related variables, types of drugs used, and route of consumption. This analysis had been suggested as appropriate in clustering large data sets with mixed attributes and allows the creation of models of clusters based on both categorical and continuous variables [30,31]. Schwarz’s Bayesian criteria determined the number of clusters. This method is known to be one of the most useful and objective selection criteria because it avoids the arbitrariness in traditional clustering techniques [32,33]. Because the solution of the cluster analysis could not be unique, the analysis was sometimes run changing the order of the cases [32]. The research team discussed the final solution according to its conceptual meaning.

Once the groups were established, differences in variables under study were assessed using Pearson’s chi square statistic for categorical variables and one-way analysis of variance (ANOVA) for continuous variables. Tukey’s HSD test was performed to compare differences among groups.

Before starting the analysis, we examined the assumptions of multivariate analysis. We found that the assumptions of independence and absence of multicollinearity of the variables were fulfilled. However, some continuous variables did not meet the normality criteria of the distribution. Nevertheless, the literature suggests that two-step cluster analysis is robust about the violations of distribution and independence criteria [32]. Furthermore, ANOVA is also robust about the violations of normality and homoscedasticity criteria [34]. Nevertheless, we checked the assessed differences with a non-parametric technique (Kruskal−Wallis).

The analyses were performed using the SPSS v.22 software.

## Results

### Participant characteristics

The study population consisted of 1,401 participants, with a mean age of 45.38 years (SD = 10.19). Table 1 shows a summary of the socio-demographic characteristics of the participants. The survey did not collect gender identity in those participants who identified themselves as transgender. Taking into account this limitation, we considered and labelled self-defined male and female participants as cisgender throughout the manuscript.

**Table 1:** Characteristics of the participants (n = 1,401).

Variables	n (%)
**Gender**	
Cis-men	1,100 (78.5)
Cis-women	280 (20)
Transgender	21 (1.5)
**Sexual orientation**	
Heterosexual	580 (41.4)
Homosexual	713 (50.9)
Bisexual	61 (4.4)
Others	28 (2)
Prefer not to answer	19 (1.4)
**Mode of transmission**	
Sexual intercourse	946 (67.5)
Sharing needles	272 (19.4)
Various practices concur	141 (10.1)
Other	42 (3)
**Educational level**	
No studies	57 (4.1)
Primary	383 (27.4)
Secondary	484 (34.6)
University degree	439 (31.4)
Other	37 (2.6)
**Work situation**	
Working	685 (48.9)
Unemployed	257 (18.3)
Retired or occupational disability	325 (23.2)
Other	134 (9.6)
**Monthly incomes**	
None	185 (13.2)
≤ 1,000 €	539 (38.5)
1,000−1,500 €	405 (28.9)
1,500−2,000 €	104 (7.4)
> 2,000 €	168 (12)
**Years since diagnosis**, mean ±SD	14.1±9.6
**Years taking ART**, mean ±SD	11.6±8.4
**Age**, mean ±SD	45.3±10.1

Note: Data provided in percentages, except where specified. ART: antiretroviral therapy. SD: standard deviation.

### Use of drugs and other substances

Alcohol was the substance most frequently consumed (86.7%). Ten per cent of the participants consumed it daily. More than half of the individuals were smokers (55%). Most of them smoked daily (84%), with an average of 13.1 cigarettes per day (SD = 8.5).

A total of 49.5% (n = 694) of participants stated to have used illicit drugs in the previous 12 months. The proportion of PLHIV who used illicit drugs was significantly higher in participants recruited from NGOs (54.9%) than from hospitals (43.8%), χ^2^ = 17.27, *p* < .0001. Overall, the vast majority of these participants had consumed illicit drugs before the HIV diagnosis (84.7%).

**Fig 2:**
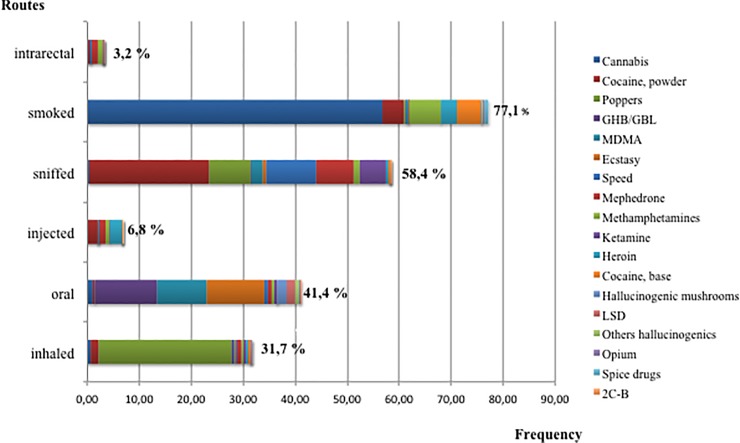
Frequencies of consumption routes used for the different types of illicit drugs. N = 694.

The most prevalent illicit drugs were cannabis, cocaine and poppers. Cannabis was also the most regularly consumed substance (Fig 1). Almost a quarter of PLHIV who used illicit drugs consumed only cannabis (24.9%). The average number of different drugs used per participant in the last 12 months was 3.3 (SD = 0.7; range: 1−14). Injected and rectal routes of administration accounted for 6.8% and 3.2% of the cases, respectively (Fig 2). Heroin, cocaine, and mephedrone were the most frequent injected drugs; mephedrone and methamphetamine the most intrarectally administered.

The use of some types of illicit drugs differed between the two biggest cities in Spain (Madrid and Barcelona). Drugs that showed more differences between these cities were poppers, ecstasy and mephedrone. In this sense, consumption of mephedrone and poppers were higher in Madrid than in Barcelona (33.3% vs. 19.8%; χ^2^ = 7.78, *p* = .005 and, 68.5% vs. 56.9%; X^2^ = 4.75, *p* = .029, respectively). Conversely, consumption of ecstasy and ketamine were higher in Barcelona than in Madrid (37.1% vs. 27.2%; χ ^2^ = 3.73, *p* = .053 and, 22.8% vs. 13.0%; χ ^2^ = 5.35, *p* = .021, respectively).

**Fig 3:**
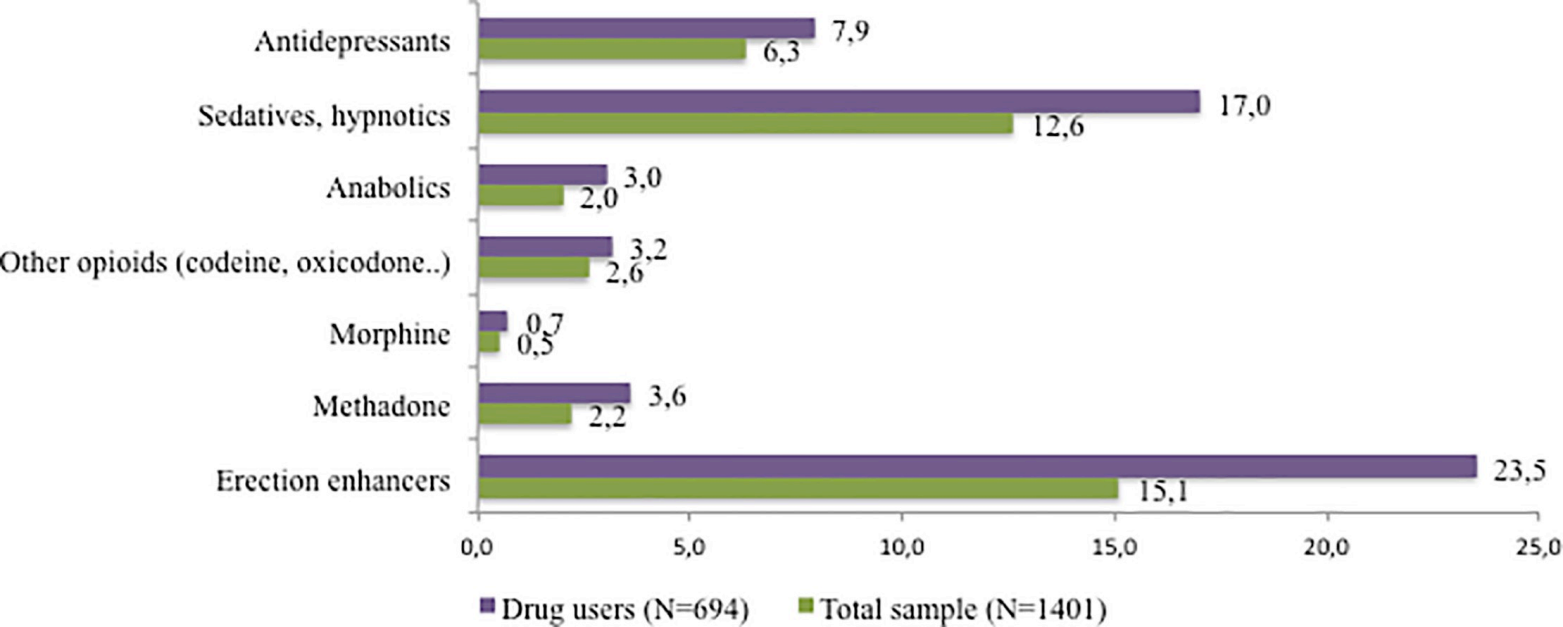
Percentage of PLHIV who used other medicines or substances.

In addition to illicit drugs, 27.1% of the total sample (n = 379) consumed other substances. PLHIV who used illicit drugs consumed them more prevalently than PLHIV who did not use illicit drugs (37.0% vs. 17.3%, χ ^2^ = 69.40; p < .0001). The most frequent substance consumed both among the total sample, and among PLHIV who used illicit drugs were erection enhancers (Fig 3).

### Patterns of use of illicit drugs

The two-stage cluster analysis classified the participants into four groups. All the variables included in the analysis contributed significantly to the formation of the groups. Table 2 summarizes the consumers’ profiles in clusters according to socio-demographic characteristics and use of drugs and other medicines or substances. Tables 3 and 4 display detailed information and statistics.

**Table 2:** Cluster’s profile according to demographics, health-related data, use of drugs and other medicines.

Characteristics	C1 (24.8%, n = 172)	C2 (12.1%, n = 84)	C3 (41.1%, n = 285)	C4 (22.0%, n = 153)
**Gender**	Mostly cis-men but the highest percentage of cis-women among clusters	Mainly cis-men but some cis-women and transgender	Mainly cis-men but some transgender	Only cis-men
**Age**	~ 50 years	~ 50 years	~ 40 years	< 40 years
**Sexual orientation**	Mainly HTX	Mainly HTX	Mainly MSM	Mainly MSM
**Educational level**	Low level	The lowest level	High level	The highest level
**Work situation**	Most retired or unemployed	Most retired and the highest percentage of unemployed	Most working	Most working, the lowest percentage of retired
**Economic status**	Low	The lowest	Middle−high	The highest
**Mode of HIV transmission**	Mainly injection	Mainly (the highest) injection	Mainly sexual	Mainly sexual
**Time living with HIV**	More than 20 years	More than 20 years	Near to 10 years	Less than 10 years
**Type of drugs used**	Mainly users of cannabis and some of them cocaine powder	Mainly users of cannabis, heroin and cocaine	Mainly users of cannabis, cocaine and poppers	The highest percentage of using most types of drugs except heroin
**Polyconsumption**	Low	Medium	Medium	High
**Routes of consumption**	Mainly smoked, some sniffed	Mainly smoked and sniffed, highest % of PWID	Mainly smoked, sniffed, inhaled and around 1/3 oral	All routes. Some PWID. Anal route
**Other medicines or substances**	Most sedatives and around 1/3 antidepressants. Some methadone and erection enhancers	Mainly sedatives, methadone or other opioids. Half of them antidepressants	Mainly erection enhancers and near to 1/3 sedatives	Most erection enhancers (the highest %), good few sedatives. The highest % of anabolic users
**Reasons for consumption**	Mainly relaxing or avoiding worries	Mainly relaxing or avoiding worries and negative feelings	Mainly to enjoy and sexual purposes	Mainly to enjoy and sexual purposes

N = 694. HTX: heterosexual; MSM: men who have sex with other men.

**Table 3:** Cluster’s profile according to socio-demographic and health data.

Variable	C1 (n = 172)	C2 (n = 84)	C3 (n = 285)	C (n = 153)	PLHIV who did not use drugs (n = 707)	Contrast statistic^a^
**Gender**^b^						χ^2^ = 186.97
Cis-men	61	78.6	96.1	100	71	
Cis-women	38.4	19	0	0	28	
Transgender	0.6	2.4	3.9	0	1	
**Sexual orientation**^b^						χ^2^ = 547.10
Heterosexual	86.6	85.7	6	0	48.4	
Homosexual	2.9	3.6	87	94.8	44.1	
Bisexual	6.4	6	4.6	3.3	3.8	
Others	1.7	1.2	2.1	2	2.1	
Prefer not to answer	2.3	3.6	0.4	0	1	
**Mode of transmission** ^b^						χ^2^ = 458.65
Sexual intercourse	31.4	16.7	87	92.2	69.2	
Sharing needles	57.6	75	0	0	15.6	
Various practices concur	9.9	6	10.2	6.5	11.3	
Other	1.2	2.4	2.8	1.3	4	
**Educational level**^b^						χ^2^ = 273.76
No studies	8.7	14.5	1.1	0	3.8	
Primary	50.6	61.4	11.6	8.5	28.1	
Secondary	31.4	13.3	40.4	29.4	36.6	
University degree	7	8.4	43.5	58.2	29.3	
Other	2.3	2.4	3.5	3.9	2.1	
**Work situation**^b^						χ^2^ = 198.77
Working	23.8	13.1	69.1	69.3	46.7	
Unemployed	22.1	34.5	16.1	20.9	15.8	
Retired or occupational disability	41.3	41.7	8.4	3.9	26.7	
Other	12.8	10.7	6.3	5.9	10.7	
**Monthly incomes**^b^						χ^2^ = 148.76
None	14.5	21.4	10.5	12.4	13.2	
≤ 1,000 €	61	60.7	23.5	17.6	40.9	
1,000−1,500 €	15.1	9.5	41.8	39.2	27.2	
1,500−2,000 €	3.5	4.8	8.8	13.1	6.9	
> 2,000 €	5.8	3.6	15.4	17.6	11.9	
**Inmunological status**						χ^2^ = 28.23
< 200 CD4 mm^*3*^	9.6	7.2	3.5	1.7	6.6	
200−400 CD4 mm^*3*^	14.4	17.4	7.1	6	15.4	
> 400 CD4 mm^*3*^	76	75.4	89.4	92.2	78	
**Undetectable viral load**	93.3	87.8	94.4	97.4	93	χ^2^ = 9.02; *p* = .061
**Years since diagnosis**^b^^,^^1^, mean ±SD	22.5±8.3	23.2±6.1	9.2±7.3	7.0±5.1	17.7±9.5	*F* = 114.36
**Years taking ART**^2^, mean ±SD	18.0±7.2	18.2±6.8	7.8±6.8	5.6±4.5	12.4±8.4	*F* = 82.09; *p* = .000
**ART adherence**^3^, mean±SD	84.1±10.5	79.8±14.3	87.4±8.7	86.3±9.1	88.3±8.5	*F* = 20.83
**Age**^b^^,^^4^, mean ±SD	50.9±6.4	49.5±5.5	41.1±9.4	36.8±8.3	47.1±10.3	*F* = 73.20
STIs*^5^ (%, (m±SD)	10.5 (1.3±0.5)	13.1 (1.4±1.2)	30.9 (1.4±0.7)	60.8 (1.81±1.1)	16 (1.4±0.8)	*F* = 3.71; *p* < .01

Note: Data provided in percentages, except where specified. ART: antiretroviral therapy. STIs: sexually transmitted infections suffered in the last year (* percentage of people in each cluster who had suffered any STI in the last year and mean of number of STIs suffered in the last year).

^a^ All differences were significant at *p* < .0001 except when it is specified in the table.

^b^ Variables included in two-stage cluster analysis.

^1^HSD Tukey results found differences between C1 and C2, C3, C4 and PLHIV who did not use illicit drugs (*p* < .0001); differences between C3 and C4 were marginally different (*p* = .069)

^2^Differences were found between C1, C2, C3, C4 and PLHIV who did not use illicit drugs (p < .0001), and between C3 and C4 (*p* < .05)

^3^Differences were found between C1 and: C2 (*p* = .005),C3 (*p* = .002) and PLHIV who did not use illicit drugs (*p* < .0001), and between C2 and C3, C4 and PLHIV who did not use illicit drugs (*p* < .0001)

^4^There were differences between all the groups (*p* < .0001) except between C1 and C2, and C2 and PLHIV who did not use illicit drugs

^5^ Significant differences were found between C4 and both C3 and PLHIV who did not use illicit drugs.

**Table 4:** Cluster’s profile according to the type and frequency of drugs used, routes of consumption and polyconsumption.

	C1 (n = 172)	C2 (n = 84)	C3 (n = 285)	C4 (n = 153)	Contrast statistic^a^
**Drugs %** (mean±SD)^b^^,^^e^					
Cannabis	86.6 (4.4±1.8)	78.6 (4.1±2.0)	63.9 (3.1±2.0)	75.2 (3.1±2.0)	χ^2^ = 30.32*** (F = 16.66***)
Cocaine (powder)	22.7 (1.6±0.9)	60.7 (2.5±1.6)	54.0 (1.6±0.9)	85.0 (1.9±1.1)	χ^2^ = 128.49***(F = 10.19***)
Cocaine (base)	0	3.6 (2.7±1.6)	0	15.7 (2.1±0.8)	χ^2^ = 75.45*** (F = .50, ns)
Heroin	0	69 (2.3±1.7)	0.4 (1.0±0.0)	0.7 (1.0±0.0)	χ^2^ = 441.50*** (F = .55, ns)
Poppers	0	0	62.5 (1.8±1.2)	89.5 (2.2±1.3)	χ^2^ = 366.59*** (F = 6.47*)
MDMA (crystal)	1.2 (1.0±0.0)	6 (1.8±1.7)	12.6 (1.0±0.2)	75.2 (1.4±0.7)	χ^2^ = 314.70*** (F = 3.24*)
MDMA (pills)	0	16.7 (3.0±2.4)	11.9 (1.0±0.2)	72.5 (1.5±0.9)	χ^2^ = 285.87*** (F = 16.99***)
Speed	4.1 (1.1±0.3)	21.4 (2.2±1.6)	8.1 (1.04±0.2)	67.3 (1.3±0.7)	χ^2^ = 249.55*** (F = 6.96***)
Methamphetamine	0.6 (1.0±0.0)	2.4 (2.0±1.4)	3.9 (1.0±0.0)	43.8 (1.5±1.0)	χ^2^ = 197.53*** (F = 1.17, ns)
GHB	0	2.4 (1.0±0.0)	19.3 (1.1±0.4)	77.1 (1.7±1.0)	χ^2^ = 305.12*** (F = 11.45***)
Mephedrone	0	0	11.6 (1.0±0.3)	62.1 (1.6±0.9)	χ^2^ = 260.60*** (F = 11.78**)
Ketamine	0.6 (1.0±0.0)	4.8 (1.0±0.0)	3.9 (1.0±0.0)	45.8 (1.4±0.8)	χ^2^ = 202.55*** (F = .97, ns)
LSD	0	8.3 (1.3±0.7)	0.7(1.0±0.0)	9.8 (1.0±0.0)	χ^2^ = 37.08*** (F = 1.24, ns)
Opium	0	7.1 (1.8±2.0)	0	2 (1.0±0.0)	χ^2^ = 28.95*** (F = .46, ns)
Spice drugs	0	2.4 (1.5±0.7)	0.4 (1.0±0.0)	2 (1.0±0.0)	χ^2^ = 6.77(ns) (F = 1.0, ns)
Mushrooms	0	3.6 (1.0±0.0)	0	15.7 (1.0±0.0)	χ^2^ = 75.45*** (—)
Other hallucinogenic plants	0	8.3 (1.0±0.0)	0.4 (1.0±0)	2.6 (1.0±0.0)	χ^2^ = 28.47*** (—)
2C-B nexus	0	1.2 (6.0±0)	0	1.3 (1.0±0.0)	χ^2^ = 5.82(ns) (—)
**Number of drugs used**^1^ (mean±SD)	1.2±0.5	3.4±2.0	2.5±1.4	7.4±2.2	F = 461.06***
**Use of drugs in sexual context%** (mean±SD) ^c^^,^^2^	3.5 (1.7±1.2)	3.6 (2.0±1.0)	38.9 (1.8±0.7)	61.4 (2.6±0.7)	F = 16.67***
**Routes of consumption**^d^^,^^e^ %					
Smoke	85.5	89.3	65.6	82.4	χ^2^ = 37.55***
Sniff	21.5	52.4	62.5	95.4	χ^2^ = 185.79***
Oral	5.2	31	35.1	98	χ^2^ = 304.21***
Injection	1.2	32.1	1.1	9.8	χ^2^ = 111.20***
Anal	0	0	0	14.4	χ^2^ = 80.33***
Inhaled	0.6	6	41.4	62.7	χ^2^ = 183.15***
**Other medicines or substances used**					
Erection enhancers	19.4	18.8	62	92.2	χ^2^ = 89.86***
Methadone	19.4	59.4	0	0	χ^2^ = 113.99***
Morphine	3.2	9.4	0	1	χ^2^ = 11.84**
Other opioids	9.7	28.1	5.4	4.9	χ^2^ = 18.59***
Anabolic	0	0	2.2	18.6	χ^2^ = 24.87***
Sedatives	61.3	93.8	30.4	40.2	χ^2^ = 42.65***
Antidepressants	32.3	50	15.2	14.7	χ^2^ = 22.54***

^a^ Measure of the importance of the categorical (χ^2^ = Pearson’s chi square) and continuous variables (one-way ANOVA) within each cluster. All χ^2^ differences were significant at *p* < .0001 except when it is specified in the table. (—) F was not calculated in drugs for which only one cluster had the variance calculated.

^b^ The frequency of consumption ranges from 1 to 6 (1: sometimes in the last 12 months; 2: once a month; 3: several times in a month; 4:once a week; 5: several times a week; 6: daily).

^c^ The frequency of the scale ranges from 1: sometimes, to 4: always.

^d^ The score of each route of consumption was calculated summing the use of the route in each drug.

^e^ Variables included in the two-step cluster analysis.

* p < .05

** p < .01

*** p < .0001.

^1^HSD Tukey results found differences between all the clusters except between C2 and C3.

^2^ HSD Tukey results found differences between CL4 and CL1 and CL3.

Two clusters were composed mostly of HTX: cluster 1 (CL1) represented about 25% of PLHIV who used illicit drugs and Cluster 2 (CL2) 12%. In these clusters, the mean age was about 50 years, and participants had been living with HIV for more than 20 years. The analysis classified most cisgender women in CL1. More than half of the participants in both clusters had completed compulsory education only, and the majority had a monthly income of less than 1,000 €. A large proportion of individuals in these clusters were retired or disabled. Sharing needles was a frequent cause of HIV acquisition in both clusters, and was the main cause in CL2. Patterns of drug use varied between these clusters. Individuals in CL1 consumed cannabis principally, followed by cocaine powder in 23% of cases. The analysis classified users of heroin in CL2, 32% of whom declared having used the injection route. Moreover, near to 60% were also taking methadone and half of them antidepressants. Polydrug use in CL2 group was higher than in CL1. The main reasons in both clusters to use drugs were ‘relaxing and avoiding worries’, 47.1% and 63.1% in CL1 and CL2, respectively, and ‘removing negative feelings’, 35.4% and 44.1% in CL1 and CL2, respectively. According to their described profile, CL1 was labelled ‘HTX mainly users of cannabis’ and CL2 ‘HTX users of heroin and cocaine’.

On the other hand, the other two clusters grouped MSM who had acquired HIV principally through sexual intercourse. Cluster 3 (CL3) represented about 42% and Cluster 4 (CL4) 22% of PLHIV using drugs. They were younger, had a higher level of education, higher socio-economic status, and a more recent HIV diagnosis compared with HTX clusters (CL1 and CL2). MSM in CL4 were the youngest participants and the ones with the most recent HIV diagnosis. Both clusters differed in their pattern of drug use. CL3 presented a moderate consumption of cannabis, poppers, and cocaine, while CL4 showed high rates of substance use for the most types of drugs and the highest polydrug use. Furthermore, almost 10% of PLHIV in CL4 used the injection route of consumption and 14.4% of them the anal route. Individuals in CL3 and CL4 clusters reported a higher use of erection enhancers’ than in HTX clusters (CL1 and CL2). The highest percentage was found among PLHIV in CL4 (92.2%). The main reasons to use drugs in both clusters (CL3 and CL4) were ‘to enjoy the substances’ (54.7% and 81.1% for CL3 and CL4, respectively) and ‘sexual purposes’ (38.9% and 61.4%, respectively). According to their described profile, CL3 was labelled ‘MSM with moderate drug use’, and CL4 ‘MSM high polydrug users’.

Almost 40% of the individuals in CL3 and about 60% in CL4 reported using drugs in a sexual context, while only around 3% in each HTX cluster used it. The frequency of use of drugs in a sexual context also differed among clusters, and was higher in CL4 than in CL3 (Table 4).

### Differences in ART adherence and other health-related variables according to the profile of use of drugs

HTX ‘users of heroin and cocaine’ (CL2) showed the lowest score in ART adherence, followed by HTX ‘mainly users of cannabis’ (CL1). MSM ‘high polydrug users’ (CL4) reported the highest percentage of STIs. MSM clusters of PLHIV who used illicit drugs (CL3 and CL4) reported the highest lymphocytes CD4 mm^*3*^ count. The lowest percentage of patients with undetectable viral load was found in CL2 (Table 3).

## Discussion

The present research revealed the prevalence of illicit drug use as well as the particular patterns of consumption among PLHIV in Spain. The most frequently consumed substances among PLHIV in Spain, according to our results, were: alcohol (86.7%), tobacco (55.0%), illicit drugs (49.5%) and other substances (27.1%). The prevalence of last-year alcohol consumption among PLHIV was higher than that reported for the general Spanish population (77.6%), although daily consumption resulted in similar results [35]. The prevalence of active smoking was high compared with the general population (30.8%) [35]. Studies in other countries focusing on PLHIV have reported similar results to those we found [36,37].

Almost half of the participants in our study had used illicit drugs in the last 12 months, with cannabis, cocaine, and poppers being the most frequent ones. Cannabis and cocaine are also the most consumed drugs in the general population [35,38] but the prevalence found in our results was three times higher for cannabis and 13 times higher for cocaine than the respective figures reported by the European Monitoring Centre for Drugs and Drug Addiction (EMCDDA) in the general population [10]. As for studies focusing on PLHIV, the prevalence found in our study was slightly higher compared with the results by Garin et al. [9]. In addition, the prevalence of cocaine use among MSM who consume drugs in our sample was higher than that found recently by González-Baeza et al. [7], but results were similar for most of the other substances, such as poppers or ketamine. At an international level, several studies have also highlighted the significant prevalence of illicit drug use in PLHIV across Europe. For example, our results were similar to those found by Daskalopoulou et al. [5] in the United Kingdom, although direct comparison is not possible since that study evaluated last-three-months consumption. Grabovak et al. [39] found a moderately higher prevalence of drug use in PLHIV in Austria but their analysis included sildenafil/tadalafil, categorized as ‘other substances’ in our study. In contrast, our prevalence results were higher compared with other studies in France [40] or Scotland [37] although, again, differences in time frame make comparisons.

Our study also found some differences in the pattern of drug use between the two largest cities in Spain (Madrid and Barcelona). Similar results were found in a study conducted in 2016 that collected data from 486 MSM in Spain, who had practised ‘chemsex’ in the previous 12 months [41]. Findings of the EMIS study showed that the prevalence of use of the different drugs varies substantially across cities, suggesting that drug use patterns appear to be culturally and socially determined [42]. The association of the city of residence with drug use could be influenced not only by psychological factors, but also by structural drivers such differences in drug availability and differences in national, regional, or local norms and cultures concerning drug use [42,43].

Regarding routes of consumption, we found that near to 7% of PLHIV who used illicit drugs were using the injection route. They represented 3.4% of the total sample. This percentage is comparable to the HIV incidence results reported in Spain and the countries from the European Union and European Economic Area [1,44].

Regarding patterns of drug use, our results found four clusters of PLHIV who used drugs. Two of them were composed mostly of HTX and the other two principally of MSM, which resulted in being the most prevalent group of PLHIV who used illicit drugs. MSM were the youngest participants, had the most recent HIV diagnosis and the best socio-economic status. These socio-demographic and epidemiologically different characteristics between MSM and HTX living with HIV are concordant with those existing in the Spanish national HIV data about HIV prevalence and incidence [1,2,45]. MSM and HTX clusters of PLHIV who used illicit drugs found in the present study also differ in their reasons for using drugs. While HTX aimed at avoiding worries or negative feelings, MSM pointed to issues such as enjoying their use and sexual purposes.

Regarding clusters of HTX who used illicit drugs, the largest one was composed of people who presented the lowest average of polydrug use and who were users of cannabis mainly followed by cocaine powder. More than a half of them were former PWID and nearly one-fifth of them were taking methadone in the period assessed by our study. Thus, they have given up or replaced the use of heroin (or other injected drugs). In addition, a small group of HTX was also found who were polydrug users and who showed the highest prevalence of heroin use and current injection route. Although the majority of PLHIV in this cluster acquired HIV infection through sharing needles, during the study period only near a third of them continued injecting drugs. Taking the previous results conjointly, we can conclude that according to previous literature [19], both the use of heroin and its intravenous use have continued to decrease in Spain. In our study, PLHIV in CL2 (mainly users of heroin and cocaine) represented around 6% of the total sample and among them, 2% were PWID. Despite this decrease, it is essential to pay attention to these groups of people because we found they presented the lowest levels of adherence to treatment [20,21]. With this regard, PLHIV who were users of heroin and cocaine presented the poorest biological markers of HIV infection. However, not only the impact of their use of drugs but also their socio-epidemiological profile could explain these results. The literature shows that PLHIV who use illicit drugs are more likely to experience problematic adherence and that they have increased age-matched morbidity compared to those who do not use drugs [46,47]. A systematic review of interventions to improve adherence and virological outcomes among PLHIV who used drugs concluded that directly administered antiretroviral therapy (DAAT) obtained the most substantial evidence of efficacy but that it is labour-intensive and costly to implement [46]. The arrival of long-acting treatments could be an opportunity for DAAT in PLHIV who use illicit drugs. Nevertheless, DAAT should be complemented with other interventions that have shown efficacy, such as cognitive behavioural therapy, motivational interviewing and peer-driven interventions [46]. Besides interventions to promote adherence, pharmacokinetic drug interactions must be adequately addressed in people who use illicit drugs [47].

Among the MSM using drugs, there was a majority that reported moderate consumption, both regarding frequency and diversity of drugs used. However, there was another pattern of use in MSM, including younger patients with the highest frequency of illicit drug use and relevant polydrug consumption. Some of them used drugs intra-rectally (around 14%) and intravenously (around 10%). PWID represented around 2% of the total MSM in our study. This percentage was lower than that found by González-Baeza et al. [7] (around 4%) and similar to the one found by Folch et al. [11] (1.4%). Prevalence of PWID in Folch et al. [11] was higher in the largest towns. Baeza et al. [7] conducted their study exclusively in MSM with HIV living in a large Spanish city (Madrid), where the use of recreational drugs and the practice of ‘slamsex’ seems to be higher than in smaller cities [11,18]. Regarding other countries, Pufall et al. [13] found that 10% of the MSM interviewed in UK practised slamming in the previous year. Although MSM clusters reported frequent use of drugs in a sexual context (32%) and high rates of STIs in the previous 12 months, the percentages were the highest in MSM polydrug users. These results are similar to those reported in the study of González-Baeza et al. [7]. Recent studies are highlighting the importance of the ‘chemsex’ phenomenon and its health consequences among those who practise it [13,48,49]. Thus it will be necessary to monitor the impact of drug use in the health and psycho-social sphere of MSM who use illicit drugs. It will also be necessary to implement coordinated and multi-dimensional strategies that reduce drug-related harm among them. Specific interventions could include: direct client service support to manage or reduce the use of drugs, health promotion activities, peer education support, training healthcare providers and drug services about new recreational drugs and their role in sexual intercourse, services to address specific psychological and psychiatric problems in MSM who practice ‘chemsex’, advice about healthy leisure alternatives, harm-reduction services such as needle exchange, among others. [41,50,51]. Moreover, several study findings suggest that MSM who combine drugs with sex are more likely to engage in high-risk sexual practices when compared with MSM who do not combine drugs with sex [43]. Thus, it will be necessary to examine and increase the use of bio-medical risk reduction interventions such as STI testing, post-exposure and pre-exposure prophylaxis to reduce the potential negative consequences of ‘chemsex’ on public health [43].

Our study has several limitations. First, its cross-sectional nature does not allow causal relationships to be established. Second, we did not use a probabilistic sampling, although the large sample size and extensive geographical data collection could mitigate this limitation. In this sense, the socio-demographic characteristics of our participants were concordant with Spanish epidemiological data for PLHIV [1]. Also, we aimed to provide a general overview of drug use, including different PLHIV subgroups in Spain. This may make difficult direct comparison with studies focusing on MSM only. However, the sample in our study involved a large proportion of MSM, similar to that found in other studies [7], and specific analysis and results are provided for this subgroup. Furthermore, we used a self-reported data collection method, and this could underestimate the prevalence of PLHIV using drugs because some people would not desire to reveal this information to their health-care providers. However, we tried to overcome this limitation through the anonymity of the survey and by balancing the data collection from hospitals and NGOs. Furthermore, another limitation was that we measured the use of substances using single items. However, it allowed us to assess the use of several substances. Moreover, other studies had concluded the utility of single screening questions to identify substance use [52]. Likewise, our survey asked about gender categories (men, women, transgender) considering them as mutually exclusive. However, many transgender people identify as men or women, not as a third gender.

## Conclusions

The present research is the largest multi-centre study assessing the current prevalence and patterns of drug use among PLHIV in Spain. Our results showed a relatively high prevalence of illicit drug use among the PLHIV in Spain. Prevalence of drug use in PLHIV was higher in MSM than in HTX. MSM were mostly users of recreational and most recent drugs. Most of them presented a moderate profile of drug consumption, although there was a specific subgroup of MSM with high polyconsumption, which was associated with a higher risk of STIs. HTX tend to use mainly cannabis, heroin or cocaine. Although the use of heroin and the injection route of consumption have decreased among them, PLHIV who still used heroin and cocaine showed the most problems to adhere to HIV treatment and the worst health status. Future studies should monitor the evolution of the patterns of drug use, and the longitudinal impact of recreational drug use in their users’ health. Furthermore, it will be necessary to design strategies that provide adequate responses to the diverse problems associated with each pattern of drug use.

## Supporting information

S1 Table Percentage of surveys collected and percentage of HIV incidence in the different Spanish regions. (DOCX)

## References

[1] Dirección General de Salud Pública Calidad e Innovación. Vigilancia Epidemiológica del VIH y SIDA en España, actualización 30 de junio de 2017. Ministerio de Sanidad Servicios Sociales e Igualdad. 2016 Available from: https://www.msssi.gob.es/ciudadanos/enfLesiones/enfTransmisibles/sida/vigilancia/InformeVIH_SIDA_2017_NOV2017.pdf.

[2] Dirección General de Salud Pública Calidad e Innovación. Encuesta Hospitalaria de pacientes con infección por el VIH: Resultados 2016. Análisis de la evolución 2001–2016. Ministerio de Sanidad, Servicios Sociales e Igualdad. 2017. Available from: https://www.msssi.gob.es/ciudadanos/enfLesiones/enfTransmisibles/sida/vigilancia/InformeEncuestaHospitalaria2016DEF.pdf.

[3] Joint United Nations Programme on HIV/AIDS(UNAIDS).UNAIDS Data 2017. Available from: http://www.unaids.org/sites/default/files/media_asset/20170720_Data_book_2017_en.pdf. 12349391

[4] Garin N, Velasco C, de Pourcq JT, Lopez B, Gutierrez MM, Haro JM, et al Recreational drug use among individuals living with HIV in Europe: review of the prevalence, comparison with the general population and HIV guidelines recommendations. Front Microbiol. 2015;6: 690 10.3389/fmicb.2015.00690 26236288 PMC4500990

[5] Daskalopoulou M, Rodger A, Phillips AN, Sherr L, Speakman A, Collins S, et al Recreational drug use, polydrug use, and sexual behaviour in HIV-diagnosed men who have sex with men in the UK: results from the cross-sectional ASTRA study. Lancet HIV. 2014;1: e22–31. 10.1016/S2352-3018(14)70001-3 26423813

[6] The EMIS Network. EMIS 2010: the European men-who-have-sex-with-men internet survey. Findings from 38 countries. Stockholm: European Centre for Disease Prevention and Control; 2013. Available from: http://www.emis-project.eu

[7] González-Baeza A, Dolengevich-Segal H, Pérez-Valero I, Cabello A, Téllez MJ, Sanz J, et al Sexualized drug use (chemsex) is associated with high-risk sexual behaviors and sexually transmitted infections in HIV-positive men who have sex withmen: data from the U-SEX GESIDA 9416 Study. AIDS Patient Care STDS. 2018;32(3): 112–118. 10.1089/apc.2017.0263 29620925

[8] Folch C, Esteve A, Zaragoza K, Muñoz R, Casabona J. Correlates of intensive alcohol and drug use in men who have sex with men in Catalonia, Spain. Eur J Public Health. 2010;20(2): 139–45. 10.1093/eurpub/ckp091 19564240

[9] Garin N, Zurita B, Velasco C, Feliu A, Gutierrez M, Masip M, et al Prevalence and clinical impact of recreational drug consumption in people living with HIV on treatment: a cross-sectional study. BMJ Open. 2017;7: e014105 10.1136/bmjopen-2016-014105 28100565 PMC5253545

[10] European Monitoring Centre for Drugs and Drug Addiction. European Drug Report. Available from: http://www.emcdda.europa.eu/system/files/publications/4541/TDAT17001ESN.pdf.

[11] Folch C, Fernández-Dávila P, Ferrer L, Soriano R, Díez M, Casabona J. Alto consumo de drogas recreativas y conductas sexuales de riesgo en hombres que tienen relaciones sexuales con hombres. Med Clin (Barc). 2015;145(3): 102–107. 10.1016/j.medcli.2014.04.030 25256434

[12] Semple SJ, Strathdee SA, Zians J, Patterson TL. Sexual risk behavior associated with co-administration of methamphetamine and other drugs in a sample of HIV-positive men who have sex with men. Am J Addict. 2009;18(1): 65–72. 10.1080/10550490802544466 19219667 PMC3044646

[13] Pufall EL, Kall M, Shahmanesh M, Nardone A, Gilson R, Delpech V, et al Sexualized drug use (“chemsex”) and high-risk sexual behaviours in HIV-positive men who have sex with men. HIV Med. 2018;19(4): 261–70. 10.1111/hiv.12574 29368440 PMC5900961

[14] Stuart D, Weymann J. ChemSex and care-planning: one year in practice. HIV Nurs. 2015;15: 24–28.

[15] Edmundson C, Heinsbroek E, Glass R, Hope V, Mohammed H, White M, et al Sexualised drug use in the United Kingdom (UK): a review of the literature. Int J Drug Policy. 2018;55: 131–148. 10.1016/j.drugpo.2018.02.002 29625796

[16] Ottaway Z, Finnerty F, Amlani A, Pinto-Sander N, Szanyi J, Richardson D. Men who have sex with men diagnosed with a sexually transmitted infection are significantly more likely to engage in sexualised drug use. Int J STD AIDS. 2017;28(1): 91–93. 10.1177/0956462416666753 27542697

[17] Stuart D. Sexualised drug use by MSM: background, current status and response. HIV Nurs. 2013; 6–10.

[18] Strathdee SA, Stockman JK. Epidemiology of HIV among injecting and non-injecting drug users: current trends and implications for interventions. Curr HIV/AIDS Rep. 2010;7(2): 99–106. 10.1007/s11904-010-0043-7 20425564 PMC2856849

[19] De la Fuente L, Brugal MT, Domingo-Salvany A, Bravo M, Neira-León M, Barrio G. Más de treinta años de drogas ilegales en España: una amarga historia con algunos consejos para el futuro. Rev Esp Salud Pública. 2006;80(5): 505–520. 17193814 10.1590/s1135-57272006000500009

[20] Dawson-Rose C, Draughon JE, Zepf R, Cuca YP, Huang E, Freeborn K, et al Prevalence of substance use in an HIV primary care safety net clinic: a call for screening. J Assoc Nurses AIDS Care. 2017;28(2): 238–249. 10.1016/j.jana.2015.12.001 26763795 PMC4903083

[21] Claborn K, Becker S, Operario D, Safren S, Rich JD, Ramsey S. Adherence intervention for HIV-infected persons who use drugs: adaptation, open trial, and pilot randomized hybrid type-1 trial protocol. Addict Sci Clin Pract. 2018;13(1): 12 10.1186/s13722-018-0113-5 29606129 PMC5879738

[22] Korthuis PT, Edelman EJ. Substance use and the HIV care continuum: important advances. Addict Sci Clin Pr. BioMed Central; 2018;13: 13 10.1186/s13722-018-0114-4 29530080 PMC5848588

[23] Staltari O, Leporini C, Caroleo B, Russo E, Siniscalchi A, Sarro G De, et al Drug−Drug interactions: Antiretroviral drugs and recreational drugs. Recent Pat CNS Drug Discov. 2014;9(3): 153–163. 25429704 10.2174/1574889809666141127101623

[24] Kumar S, Rao P, Earla R, Kumar A. Drug–drug interactions between anti-retroviral therapies and drugs of abuse in HIV systems. Expert Opin Drug Metab Toxicol. 2015;11(3): 343–55. 10.1517/17425255.2015.996546 25539046 PMC4428551

[25] Cashman SB, Adeky S, Allen AJ, Corburn J, Israel BA, Montaño J, et al The power and the promise: working with communities to analyze data, interpret findings, and get to outcomes. Am J Public Health. 2008;98(8): 1407–17. 10.2105/AJPH.2007.113571 18556617 PMC2446454

[26] Wallerstein NB, Duran B. Using community-based participatory research to address health disparities. Health Promot Pract. 2006;7(3): 312–323. 10.1177/1524839906289376 16760238

[27] Revicki DA, Gnanasakthy A, Weinfurt K. Documenting the rationale and psychometric characteristics of patient reported outcomes for labeling and promotional claims: the PRO evidence dossier. Qual Life Res. 2007;16(4): 717–723. 10.1007/s11136-006-9153-5 17268927

[28] Eignor DR. Standards for the development and use of tests: the standards for educational and psychological testing. Eur J Psychol Assess. 2001;17(3): 157–163. 10.1027//1015-5759.17.3.157

[29] Remor E. Systematic review of the psychometric properties of the questionnaire to evaluate the adherence to HIV therapy (CEAT‐VIH). Patient. 2013;6(2): 61–73. 10.1007/s40271-013-0009-0 23558754

[30] Chiu T, Fang D, Chen J, Wang Y, Jeris C. A robust and scalable clustering algorithm for mixed type attributes in large database environment. In: KDD ‘01: Proceedings of the Seventh ACM SIGKDD International Conference on Knowledge Discovery and Data Mining. San Francisco, California: ACM; 2001 pp. 263–268. 10.1145/502512.502549

[31] Norušis MJ. SPSS 12.0 statistical procedures companion. Chicago:ed. Prentice Hall; Upper Saddle River, NJ; 2004.

[32] Pérez C. Técnicas de análisis multivariante de datos. aplicaciones con SPSS®. Madrid: ed. Prentice Hall; 2004.

[33] Okazaki S. What do we know about mobile Internet adopters? A cluster analysis. Inf Manag. 2006;43(2): 127–141. 10.1016/j.im.2005.05.001

[34] Lix LM, Keselman JC, Keselman HJ. Consequences of assumption violations revisited: a quantitative review of alternatives to the one-way analysis of variance F-test. Rev Educ Res. 1996;66(4): 579–619. 10.3102/0034653066004579

[35] Observatorio Español de Drogas y Adicciones. Informe 2017: Alcohol, Tabaco y Drogas ilegales en España. Ministerio de Sanidad Servicios Sociales e Igualdad. Available from: http://www.pnsd.mscbs.gob.es/profesionales/sistemasInformacion/informesEstadisticas/pdf/2017OEDA-INFORME.pdf

[36] Williams EC, Hahn JA, Saitz R, Bryant K, Lira MC, Samet JH. Alcohol use and human immunodeficiency virus (HIV) infection: current knowledge, implications, and future directions. Alcohol Clin Exp Res. 2016;40(10): 2056–2072. 10.1111/acer.13204 27696523 PMC5119641

[37] Li J, Mcdaid LM. Alcohol and drug use during unprotected anal intercourse among gay and bisexual men in Scotland: what are the implications for HIV prevention? Sex Transm Infect. 2014;90(2): 125–132. 10.1136/sextrans-2013-051195 24345556 PMC3932751

[38] European Monitoring Centre for Drugs and Drug Addiction. Spain Drug Report 2018. Available from: http://www.emcdda.europa.eu/countries/drug-reports/2018/spain_en.

[39] Grabovac I, Meilinger M, Schalk H, Leichsenring B, Dorner TE. Prevalence and associations of illicit drug and polydrug use in people living with HIV in Vienna. Sci Rep. 2018;8(1): 8046 10.1038/s41598-018-26413-5 29795303 PMC5966416

[40] Allavena C, Guimard T, Billaud E, de la Tullaye S, Reliquet V, Pineau S, et al Prevalence and risk factors of sleep disturbances in a large HIV-infected adult population. J Int AIDS Soc. 2014;17: 19576 10.7448/IAS.17.4.19576 25394083 PMC4224925

[41] Zaro I, Navazo T, Vázquez J, García A, Ibarguchi L. A closer look at chemsex in Spain, 2016. Imagina Más. Apoyo Positivo. Avalaible from: https://apoyopositivo.org/wp-content/uploads/2017/04/A-closer-look-at-Chemsex-in-Spain-2016.pdf.

[42] Schmidt AJ, Bourne A, Weatherburn P, Reid D, Marcus U, Hickson F, et al Illicit drug use among gay and bisexual men in 44 cities: Findings from the European MSM Internet Survey (EMIS). Int J Drug Policy. 2016;38:4–12. 10.1016/j.drugpo.2016.09.007 27788450

[43] Maxwell S, Shahmanesh M, Gafos M. Chemsex behaviours among men who have sex with men: A systematic review of the literature. Int J Drug Policy. 2019;63:74–89. 10.1016/j.drugpo.2018.11.014 30513473

[44] Joint United Nations Programme on HIV/AIDS (UNAIDS). UNAIDS DATA 2018. Available from: http://www.unaids.org/sites/default/files/media_asset/unaids-data-2018_en.pdf. 12349391

[45] Fuster-Ruiz de Apodaca MJ, Arazo P, López JC, Sánchez N, Cotarelo M, Dalmau D. HIV patients ‘ appraisal of antiretroviral treatment characteristics in Spain. Rev Multidiscip del SIDA. 2015;3(5): 7–20.

[46] Binford MC, Kahana SY, Altice FL. A systematic review of antiretroviral adherence interventions for HIV-infected people who use drugs. Curr HIV/AIDS Rep. 2012;9:287–312. 10.1007/s11904-012-0134-8 22936463 PMC3495269

[47] Altice FL, Kamarulzaman A, Soriano V V., Schechter M, Friedland GH. Treatment of medical, psychiatric, and substance-use comorbidities in people infected with HIV who use drugs. Lancet. 2010;376:367–87. 10.1016/S0140-6736(10)60829-X 20650518 PMC4855280

[48] Glynn RW, Byrne N, O ‘Dea S, Shanley A, Codd M, Keenan E, et al Chemsex, risk behaviours and sexually transmitted infections among men who have sex with men in Dublin, Ireland. Int J Drug Policy. 2018;52: 9–15. 10.1016/j.drugpo.2017.10.008 29223761

[49] Coll J, Fumaz CR. Drogas recreativas y sexo en hombres que tienen sexo con hombres: chemsex. Riesgos, problemas de salud asociados a su consumo, factores emocionales y estrategias de intervención. Rev Enf Emerg. 2016;15(2): 77–84.

[50] Ramos C, Pardo L, Clara CG, Prado M, Soler V. Programa de educación y salud sexual para usuarios de chemsex: una respuesta coordinada desde la ONG Apoyo Positivo y el Instituto de Adicciones de Madrid. Rev Multidiscip del Sida. 2019;7(16):48–52.

[51] Stardust Z, Kolstee J, Joksic S, Gray J and Hannan S. A community-led, harm-reduction approach to chemsex: case study from Australia’s largest gay city. Sex Health. 2018;15:179–81. 10.1071/SH17145 29592830

[52] Saitz R, Cheng DM, Allensworth-Davies D, Winter MR, Smith PC. The ability of single screening questions for unhealthy alcohol and other drug use to identify substance dependence in primary care. J Stud Alcohol Drugs. 2014;75(1): 153–157. 10.15288/jsad.2014.75.153 24411807 PMC3893629

