# Supplementary material for: Prevalence and patterns of illicit drug use in people living with HIV in Spain: A cross-sectional study
Source: PLoS One. 2019 Jun 17;14(6):e0211252. doi: 10.1371/journal.pone.0211252 (PMC6576760; doi:10.1371/journal.pone.0211252)
Supplement: S1 Table — (DOCX) [file pone.0211252.s001.docx]

**S1 Table. Percentage of surveys collected and percentage of HIV incidence in the different Spanish regions.**

| **Regions** | **% Surveys** (n) | **% of accumulated HIV**  **incidence 2013-2017** (n) |
| --- | --- | --- |
| Cataluña | 24.5 (343) | 17.02 (3,425) |
| Madrid | 22.7 (318) | 23.3 (4,698) |
| Andalucía | 19.3 (271) | 16.2 (3,271) |
| Comunidad Valenciana | 13.4 (189) | 9.9 (2,000) |
| País Vasco | 3.6 (51) | 3.7 (738) |
| Murcia | 3.4 (48) | 2.9 (591) |
| Galicia | 3.3 (46) | 3.8 (769) |
| Islas Baleares | 2.8 (39) | 3.9 (783) |
| Aragón | 2.4 (34) | 2.6 (536) |
| Asturias | 1.5 (21) | 1.7 (348) |
| Castilla y León | 1.5 (21) | 2.5 (519) |
| Cantabria | 1.4 (20) | 1.1 (228) |
| Total | 1,401 | 20,124 |
